# Case Report: Neuroleptic malignant syndrome in a 15-year-old female following of psychotropic drugs

**DOI:** 10.3389/fpsyt.2025.1560248

**Published:** 2025-05-20

**Authors:** Zhao-kun Fan, Ru-qin Yi, Zhi-rong Zhang, Ying-ying Shen

**Affiliations:** ^1^ The Second School of Clinical Medicine, Zhejiang Chinese Medical University, Hangzhou, China; ^2^ Department of Intensive Care Unit, The First Affiliated Hospital of Zhejiang Chinese Medical University, Zhejiang Provincial Hospital of Chinese Medicine, Hangzhou, China; ^3^ Department of Medical Record, The First Affiliated Hospital of Zhejiang Chinese Medical University, Zhejiang Provincial Hospital of Chinese Medicine, Hangzhou, China

**Keywords:** neuroleptic malignant syndrome, antipsychotic overdose, rhabdomyolysis, vocal cord dysfunction, plasmapheresis

## Abstract

**Background:**

Neuroleptic Malignant Syndrome (NMS) is a rare but life-threatening neurological emergency associated with dopamine receptor blockade. It is characterized by hyperthermia, autonomic instability, muscle rigidity, and altered mental status. Early recognition and intervention are crucial to prevent severe complications.

**Case presentation:**

We report a case of a 15-year-old female diagnosed with major depressive disorder (MDD), with a body mass index (BMI) of 13.7, who developed severe NMS with rapid-onset rhabdomyolysis and vocal cord dysfunction following an overdose of quetiapine, sertraline, and lorazepam. The clinical presentation posed a diagnostic challenge due to overlapping features with serotonin syndrome. Despite aggressive supportive care, she developed acute kidney injury secondary to rhabdomyolysis, necessitating plasmapheresis and hemoperfusion. Additionally, post-extubation vocal cord dysfunction led to aspiration pneumonia, requiring prolonged airway management.

**Conclusion:**

This case highlights the complexity of diagnosing NMS in the context of polypharmacy, the potential for rapid rhabdomyolysis, and the rarity of vocal cord involvement. The successful use of plasmapheresis as an adjunct therapy suggests a possible role in severe NMS with organ dysfunction. Early recognition and individualized management remain key to optimizing outcomes.

## Introduction

Neuroleptic Malignant Syndrome (NMS) is a severe and potentially fatal condition commonly associated with the use of dopamine receptor antagonists or the abrupt discontinuation of dopaminergic medications (1). It is characterized by altered mental status, muscle rigidity, hyperthermia, and autonomic dysfunction, requiring urgent medical intervention. Although nearly all antipsychotic medications have been implicated in NMS, its incidence remains low. However, due to its high mortality risk and severe clinical outcomes, prompt recognition and management are essential (2). The cornerstone of NMS treatment involves the immediate discontinuation of the offending drug and aggressive supportive care to prevent complications. In severe cases, pharmacological interventions such as bromocriptine or levodopa-carbidopa may be required to restore dopaminergic transmission. Since its first reports in the 1960s, the mortality rate of NMS has decreased from 76% to 10–20%, likely due to increased awareness, earlier diagnosis, and more effective treatment strategies (3). Despite its rarity, a high index of clinical suspicion is necessary for diagnosing NMS. The incidence among patients receiving antipsychotic agents is estimated to range from 0.02% to 3%. NMS can occur after a single dose or prolonged treatment, and while it is not strictly dose-dependent, higher doses are considered a risk factor (4, 5). Additionally, the clinical spectrum of NMS is highly variable, and some cases may present with severe complications such as rhabdomyolysis or, in rare instances, vocal cord paralysis. This report describes a case of a 15-year-old female who developed NMS following an overdose of antipsychotic medications, complicated by an unusual presentation of glottic dysfunction. The clinical features and therapeutic approaches of this case are discussed to enhance the understanding and management of NMS.

## Case presentation

A 15-year-old adolescent female was admitted to the emergency department of Zhejiang Provincial Hospital of Traditional Chinese Medicine, Qiantang District, on June 13, 2023, 14 hours after overdosing on quetiapine (350mg), sertraline (700mg), and an unspecified dose of lorazepam. She had a two-year history of depression and had been on a stable regimen of the aforementioned medications: quetiapine extended-release formulation 50 mg daily, sertraline 100 mg daily, and lorazepam 1 mg daily. The patient’s family reported that she ingested an excessive amount of the aforementioned drugs around 10:00 pm on June 12th. At approximately 4:00 am on June 13th, she began to experience hallucinations and delirium, reporting seeing people who were not present and hearing indistinct voices, which caused significant distress and fear. These symptoms were accompanied by an unsteady gait, nausea, vomiting, headache, and dizziness. By 10:00 am, she developed limb tremors. The family sought further treatment and transported her to our emergency department.

**Figure 1:**
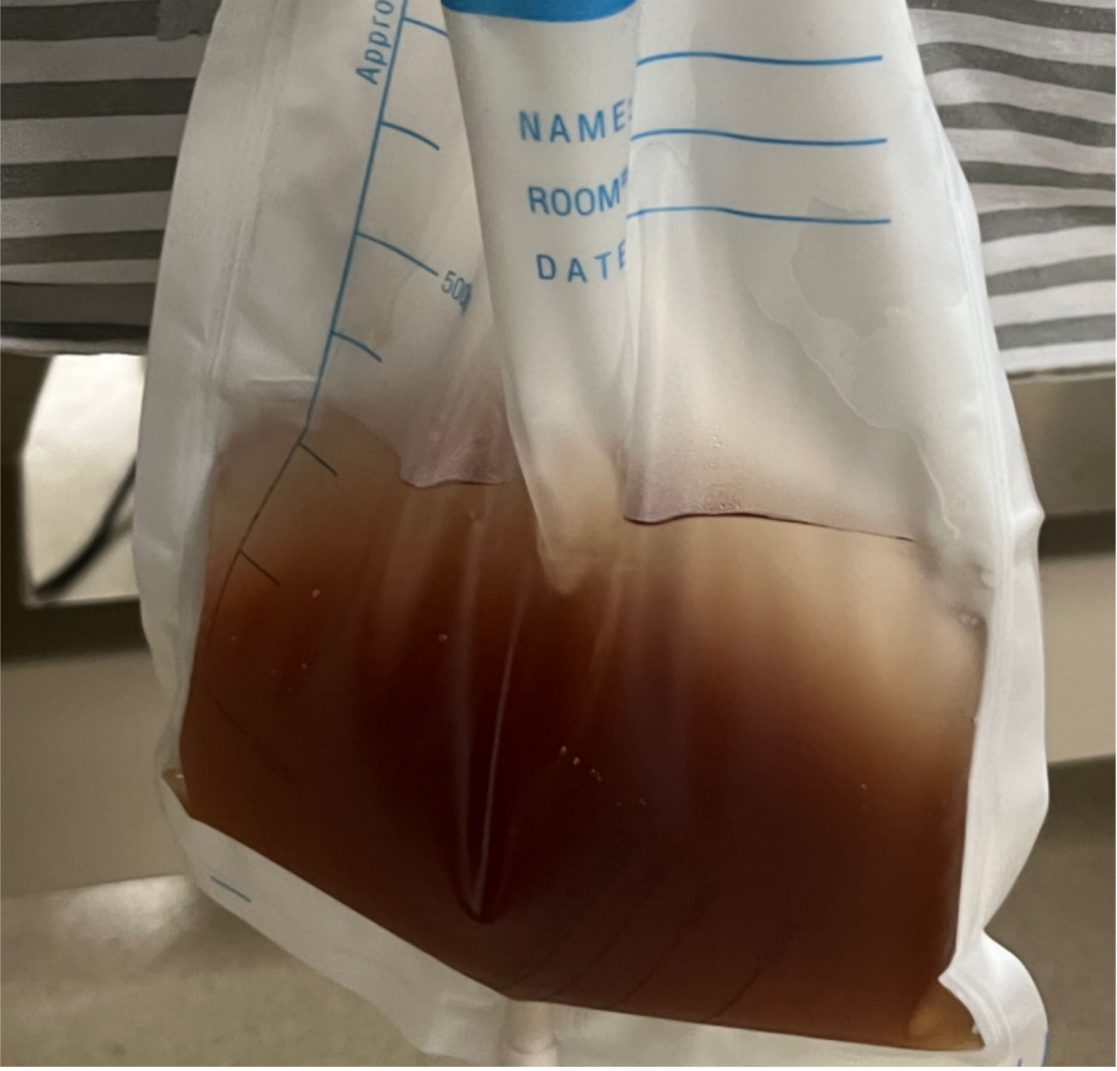
The patient’s urine appeared dark brown, which raised strong suspicion for rhabdomyolysis.

Upon presentation to the emergency department, the patient was in a delirious state with hallucinations and displayed inappropriate responses. Her Glasgow Coma Scale (GCS) score was 13 (E4, V3, M6), indicating mild impairment of consciousness. She had a mild fever with a temperature of 38.2°C, a heart rate of 106 beats/min, regular rhythm, respiratory rate of 20 breaths/min, and a blood pressure of 126/72mmHg. She stood at 155 cm, appeared emaciated, and weighed 33 kg. Her calculated BMI was 13.7, indicating severe underweight. There was no jaundice on skin or mucous membranes, no superficial lymphadenopathy, her neck was supple without resistance. Lung auscultation revealed clear breath sounds with no rhonchi. The abdomen was flat with bowel sounds at 2–3 times/min. Limb tremors were present with increased muscle tone. Bilateral Babinski sign was negative, but ankle clonus was positive. Emergency lab tests revealed a significant elevation in creatine kinase (as shown in **Table 1**). Her urine was dark brown (as shown in **Figure 1**).

**Table 1:** Laboratory test results.

Variable	Reference Range, Adults	On Arrival, This Hospital	On Arrival at ICU, 3 Hr after Arrival, This Hospital	1 day after Arrival, This Hospital
Hemoglobin (g/dl)	11.5-15.0	12.5	11.3	10.7
Platelet count (per μl)	125,000–350,000	277,000	245,000	146,000
White−cell count (per μl)	3,500–9,500	15,100	11,900	7,700
Sodium (mmol/liter)	137–147	140	140	147
Potassium (mmol/liter)	3.5–5.3	3.9	3.7	3.8
Creatinine (umol/liter)	45-84	68	60	30
Glucose (mmol/liter)	3.89-6.11	6.41	6.53	6.05
Total protein (g/dl)	6.5–8.5	7.7	6.1	5.3
Albumin (g/dl)	4.0–5.5	4.7	4.0	3.4
Total bilirubin (umol/liter)	3.4-20.5	25.3	18.8	13.1
Prothrombin time (sec)	9.8-14.0	13.2	14.2	13.6
Partial−thromboplastin time (sec)	25.0-36.0	24.1	25.7	30.6
Troponin T (ug/liter)	0.000-0.026	0.002	0.005	0.02
Creatine kinase (U/liter)	40-200	18127	20930	3165
Creatine kinase MB isoenzyme (U/liter)	0.0-24.0	123.2	115.9	10.9

Patient Publication Consent, Next of Kin Consent Obtained.

**Figure 2:**
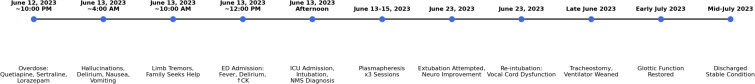
Medical history and clinical course.

Given her history of overdose with psychotropic medications and the presence of fever and rhabdomyolysis, a diagnosis of NMS was made. She was admitted to the Intensive Care Unit (ICU) for intubation and mechanical ventilation, as well as plasmapheresis and hemoperfusion. Over the course of her stay, she underwent three sessions of plasmapheresis (once daily for three consecutive days). Ten days post-admission (June 23rd), her condition improved. Limb tremors subsided, and she became alert. After evaluating her oxygenation and respiratory function, the endotracheal tube was removed. However, upon tube removal, the patient experienced glottis closure failure, resulting in aspiration and decreased oxygenation. Consequently, tracheal intubation was reinstated. Over the subsequent week, a tracheostomy was performed, and the patient was successfully weaned from the ventilator. Following swallow and speech rehabilitation training, the patient’s glottis function was restored, enabling independent vocalization and eating. Eventually, the tracheostomy tube was successfully removed. The patient received regular follow-up from the mental health department and was discharged without complications. (The patient’s medical history and clinical course are illustrated in **Figure 2**).

## Discussion

NMS is a life-threatening neurological emergency associated with antipsychotic use, characterized by altered mental status, muscle rigidity, fever, and autonomic dysfunction (6, 7). Due to its unpredictable occurrence and potential for fatal systemic complications, early recognition and management are critical. NMS has been observed with all classes of antipsychotics, including low-potency agents (e.g., chlorpromazine) and second-generation antipsychotics (e.g., clozapine, risperidone, olanzapine), as well as antiemetics (e.g., metoclopramide, promethazine, levosulpiride) (8), its risk is influenced by rapid dose escalation, drug alteration, and non-oral administration (9–12).

Mechanistically, NMS results from dopamine D2 receptor blockade, leading to autonomic dysregulation, hyperthermia, and sustained muscle rigidity, predisposing patients to rhabdomyolysis and multi-organ dysfunction. CK elevation is a hallmark of NMS, often exceeding 1000 IU/L, with peak levels correlating with disease severity (13–15). While CK levels typically rise gradually, this patient demonstrated rapid and severe rhabdomyolysis shortly after admission, suggesting an accelerated disease course.

One possible explanation is the pharmacokinetic interaction between sertraline and quetiapine, which approved by FDA for the treatment of depression in adults. Sertraline is a CYP3A4 inhibitor, and quetiapine is metabolized via CYP3A4 (16); thus, their combination may have prolonged quetiapine clearance, intensified dopamine blockade, and exacerbated muscle rigidity, contributing to faster rhabdomyolysis progression. While rhabdomyolysis is well-documented in NMS, such rapid onset is uncommon, particularly in adolescent patients, warranting further investigation into drug interactions and individual susceptibility factors.

This case highlights the diagnostic complexity of NMS, particularly in the context of polypharmacy and overlapping syndromes. The differential diagnosis included Serotonin Syndrome (SS), Hemolytic Uremic Syndrome (HUS), and Thrombotic Thrombocytopenic Purpura (TTP), and Pernicious Catatonia (PC), each of which shares features such as autonomic instability, altered sensorium, and multi-organ involvement. HUS/TTP were ruled out due to the absence of microangiopathic hemolytic anemia, thrombocytopenia, or renal failure, which are characteristic of these conditions. PC, though similar in presentation, was considered in the differential due to its association with psychiatric disorders, but was excluded given the patient’s medication history (long-term use of diazepam) and the absence of primary psychiatric symptoms. SS was differentiated based on neuromuscular findings. SS typically presents with hyperreflexia, clonus, and myoclonus, whereas NMS is characterized by lead-pipe rigidity and bradyreflexia. Additionally, NMS progresses gradually over 1–3 days, whereas SS manifests rapidly, often within hours (17–19). The presence of severe generalized rigidity, and significantly elevated CK levels favored NMS over SS in this case.

A notable feature of this case was post-extubation vocal cord dysfunction, which led to aspiration pneumonia. While autonomic instability and neuromuscular rigidity in NMS could contribute to laryngeal dysfunction, prolonged intubation-related trauma is another potential factor. The timing of post-extubation airway compromise suggests an underlying neuromuscular etiology rather than transient intubation-related spasm, making this an unusual but clinically significant airway complication of NMS.

Standard NMS management involves withdrawal of the offending drug, aggressive supportive care, and pharmacologic treatment (e.g., bromocriptine or levodopa-carbidopa). However, in this case, plasmapheresis and hemoperfusion were initiated early due to severe rhabdomyolysis and possible impending acute kidney injury (AKI). Although not a standard therapy for NMS, plasmapheresis may facilitate toxin clearance, inflammatory mediator removal, and cytokine modulation, particularly in refractory or multisystem-complicated cases.

## Conclusion

This case highlights the complexity of diagnosing NMS in the context of polypharmacy, the potential for rapid rhabdomyolysis, and the rarity of vocal cord involvement. The successful use of plasmapheresis as an adjunct therapy suggests a possible role in severe NMS with organ dysfunction. Early recognition and individualized management remain key to optimizing outcomes.

## Data Availability

The original contributions presented in the study are included in the article/**Supplementary Material**. Further inquiries can be directed to the corresponding author.
